# The present status and future growth of maintenance in US manufacturing: results from a pilot survey

**DOI:** 10.1051/mfreview/2016005

**Published:** 2016

**Authors:** Xiaoning Jin, David Siegel, Brian A. Weiss, Ellen Gamel, Wei Wang, Jay Lee, Jun Ni

**Affiliations:** 1 Department of Mechanical Engineering, University of Michigan, 360 Huntington Ave, Boston, MA 02115, USA; 2 Department of Mechanical & Materials Engineering, University of Cincinnati, 560 Baldwin Hall, 2600 Clifton Avenue, Cincinnati, OH 45221, USA; 3 U.S. Department of Commerce, National Institute of Standards and Technology, 100 Bureau Drive, Stop 8230, Gaithersburg, MD 20899, USA; 4 2300 Hayward St., Ann Arbor, MI 48109, USA

**Keywords:** Maintenance strategy, Prognostics and health management, Preventive and predictive maintenance

## Abstract

A research study was conducted (1) to examine the practices employed by US manufacturers to achieve productivity goals and (2) to understand what level of intelligent maintenance technologies and strategies are being incorporated into these practices. This study found that the effectiveness and choice of maintenance strategy were strongly correlated to the size of the manufacturing enterprise; there were large differences in adoption of advanced maintenance practices and diagnostics and prognostics technologies between small and medium-sized enterprises (SMEs). Despite their greater adoption of maintenance practices and technologies, large manufacturing organizations have had only modest success with respect to diagnostics and prognostics and preventive maintenance projects. The varying degrees of success with respect to preventative maintenance programs highlight the opportunity for larger manufacturers to improve their maintenance practices and use of advanced prognostics and health management (PHM) technology. The future outlook for manufacturing PHM technology among the manufacturing organizations considered in this study was overwhelmingly positive; many manufacturing organizations have current and planned projects in this area. Given the current modest state of implementation and positive outlook for this technology, gaps, future trends, and roadmaps for manufacturing PHM and maintenance strategy are presented.

## 1. Overview

Up-time improvement, waste reduction, and quality optimization are three important metrics for manufacturing industries to track and improve to enhance their capability and competiveness. To realize these objectives, manufacturing industries have developed several methods to evaluate manufacturing processes and systems. For example, Nakajima proposed the Overall Equipment Effectiveness (OEE) to evaluate the utilization rate or efficiency of factory equipment [1]. Since its inception, OEE has been widely adopted to evaluate factory performance. Equipment precision and process health condition are highly related to OEE, hence, there has been an increasing interest in developing intelligent maintenance systems to maintain or improve OEE in order to effectively access equipment health condition and eventually predict and prevent unwanted degradation and failures.

The increasing complexity of manufacturing equipment and processes has forced maintenance to evolve perhaps more than any other management discipline. Various maintenance strategies have been developed, such as reactive maintenance (RM), preventive maintenance (PM), and condition-based maintenance (CBM). Improvements in network communication, sensors, computing power, and machine automation have made real-time prognostic devices, remote monitoring, and self-maintenance emerging research topics on PHM technologies for manufacturing. Despite increased interest in prognostics and increased sophistication in maintenance, manufacturers lack a standard process and methodology for using PHM technologies on the shop floor.

The first step in developing a standard process and methodology for PHM technology is to understand and define a common set of performance metrics for productivity, maintenance, and product quality that are being used by manufacturers. This information allows one to quantitatively measure the effectiveness of the various diagnostic, prognostic, and intelligent maintenance activities under the same performance measures. It is also important to understand the best practices in industry for achieving maintenance and productivity goals. Surveying different manufacturers can help identify these best practices as well as less effective strategies.

### 1.1. Research objectives

The primary goal of this study is to investigate and define the best practices used by United States (US) manufacturers to improve their performance by incorporating intelligent maintenance technologies and strategies into current practice.

The main objectives of this pilot survey are:

- survey the common metrics used by the manufacturing industry to assess their productivity, maintenance and reliability, and product quality;
- research the best practices that manufacturers are using to improve their productivity, lower their maintenance costs, and improve their product quality;
- assess the current state of the art in the manufacturing sector with respect to diagnostic and prognostic activities, and review their past successes and failures.

Overall, the results of the survey-based study will provide a proper foundation for developing a set of standards and methodology for developing and implementing intelligent maintenance systems technology within manufacturing operations. The outcomes of these defined research objectives will be used to help direct the future research direction and goals for developing a unified methodology for intelligent maintenance systems for manufacturing systems.

The results from this study could determine several important aspects, including (1) whether there is a statistical difference between the number of successful implementations of diagnostic activities for large manufacturers when compared with small and medium-sized manufacturers and (2) developing an understanding of the common challenges for manufacturers for implementing prognostic and diagnostic technology. The reporting of these key findings and statistical results would be imperative for understanding the current status and needs of the manufacturing industry, and would be later used to develop appropriate standards for prognostic and diagnostic activities that address the identified needs in this survey.

### 1.2. Literature review

Table 1 presents the historical evolution and future development of maintenance practice. The following subsections provide greater detail for each maintenance strategy in Table 1.

Reactive maintenance (RM) is a corrective action applied on observable failures or unanticipated threats of failures. RM has relatively low initial cost while it may increase the costs of unscheduled equipment downtime and production losses. RM is not recommended if a failure can endanger personnel, interrupt production, or cause collateral damage.

Preventive maintenance (PM) involves the repair, replacement, and maintenance of equipment to avoid unexpected failure during operation. The objective of any PM program is the minimization of the total cost of inspection, repair, and equipment downtime (measured in terms of lost production capacity or reduced product quality). Even a successful PM strategy that improves equipment availability has two drawbacks: (1) time-based or operation count-based PM programs lead to possible under-maintained or over-maintained equipment, especially in instances when the PM interval is predetermined without considering various operation regime shifts; and (2) replacing the component before it severely degrades or fails does not allow for insightful information to be learned about the equipment’s lifecycle [2].

These drawbacks do not make PM the most cost effective program option. Eventually, preventive maintenance becomes a major expense for many industrial companies. Therefore, more efficient maintenance approaches, such as predictive maintenance (PdM) are being implemented.

Predictive Maintenance (PdM) is a right-on-time maintenance strategy. It is based on the failure limit policy in which maintenance is performed only when the failure rate or other reliability indices of a unit reaches a predetermined level. PdM can be classified into reliability-centered maintenance (RCM) and condition-based maintenance (CBM). However, this maintenance strategy has been implemented as CBM in most production systems where certain performance indices are periodically [3, 4] or continuously monitored [5]. CBM is a technique or a process for monitoring the operating characteristics of machines (or components). Changes and trends in the monitored characteristics can be used to predict the need for maintenance before serious deterioration or breakdown occurs. Thus, CBM attempts to avoid unnecessary maintenance tasks by taking maintenance actions only when there is evidence of abnormal behavior in a piece of equipment or process. By reducing the number of unnecessary scheduled preventive maintenance operations, a properly established and effectively implemented CBM program can significantly reduce maintenance costs [6, 7]. For example, based on a high-level analysis of the automotive industry, Barajas and Srinivasa [8] stated that the best return on investment (ROI) is achieved through predictive maintenance as opposed to reactive or preventive maintenance.

Prognostics and Health Management (PHM) focuses on understanding the failure modes, detecting precursors to failure, tracking degradation mechanisms, and predicting the remaining useful life of components and systems. While effective to a certain degree, neither preventive nor predictive maintenance is geared to detect the most common and subtle failure causes, such as contamination or leakage. PHM can be used to determine the root causes of failures, predict degradation trends, and take corrective actions to eliminate the sources of failure before problems occur.

In summary, the decisions to implement a proper maintenance program must be based on the probability and magnitude of the failure along with the associated costs and consequences. Designing an effective and efficient maintenance strategy requires engineering efforts that optimize the relationship between equipment ownership and operating profits by balancing cost of maintenance with cost of equipment degradation and failures and associated production losses. PdM and PHM usually require a higher maintenance cost due to higher requirement for technology readiness, but can substantially save unnecessary failures and extend the life of equipment than can simple RM and PM.

#### 1.2.1. Manufacturing prognostics and health management

Manufacturing-related prognostic and health management (PHM) research work is divided into machine-level and system-level studies, which highlights the greater emphasis found in the literature on machine-level PHM. Much of the machine-level research focuses on machine tools, including the machine tool spindle [9], cutting tool wear or breakage [10–12], and the machine tool feed-axis system [13–15]. For machine tool applications, the PHM algorithms used by researchers included both data-driven and first-principle methods, however, data-driven classification algorithms [10, 12, 13, 16] were more popular than first-principle methods. Algorithms, such as support-vector machines (SVM), self-organization maps (SOM) and variations on neural networks, and fuzzy-based diagnosis systems were some of the classification methods used in these machine tool studies. Model-based methods for machine tools were also considered by researchers, including the work by Cao et al. [9]. Cao et al. [9] used a first-principle model of the spindle to determine the optimal sensor location for spindle bearing health monitoring. Without a physical model, one might incorrectly place an accelerometer that has a less-than-desirable transfer path with respect to the fault location, which could reduce the sensitivity of the monitoring system to a bearing-related failure. Some challenges in implementing machine-level PHM in production factories are still unresolved, including how to automatically update the health models due to maintenance activities and obtaining sufficient data in a factory to validate machine-level PHM models.

For manufacturing system-level PHM, the work by Muthiah et al. [17] introduced a new metric called overall throughput effectiveness, which can provide a beneficial way to benchmark the factory’s current performance with a baseline number. Besides offering a way to monitor and trend the factory performance, this proposed metric offers promise for detecting factory bottlenecks, which would help diagnose a resultant drop in factory performance. Even for conventional metrics, such as Overall Equipment Effectiveness (OEE), it was noted that successful use of OEE depends on the ease of collecting the data and providing human-consumable visualization [18]. This sample of PHM manufacturing studies highlights the gaps in system-level diagnostics and prognostics work.

## 2. Methodology

### 2.1. Survey questionnaire development

The survey questionnaire was designed to cover a broad range of manufacturing industry sectors. The questions were designed based on different perspectives of maintenance practices and divided into five parts:

1. performance metrics for productivity, intelligent maintenance, and product quality;
2. maintenance strategy and effectiveness;
3. key factors that affect maintenance performance;
4. common problems, failure modes, and bottlenecks for manufacturing process and equipment;
5. future TRENDS for PHM technology for smart manufacturing from an industrial perspective.

The questionnaire contained questions pertaining to these five parts. All questionnaire items were operationalized using several categorical, ordinal questions and interval questions, a well-accepted practice in survey. Many of the respondents used subjective measures based on their daily observation and estimation. Although the use of objective measures would have been more desirable, it has been difficult to acquire exact data for a variety of reasons (e.g., limited data collection capability, confidentiality, accounting conventions, etc.)

### 2.2. Sample and data collection

The data were solicited via questionnaires, phone interviews, and on-site facility visits with a variety of manufacturing enterprises ranging in size. A total of 15 manufacturing enterprises and eight technology/consulting companies provided responses to the questions through surveys and interviews with manufacturing managers, maintenance managers, and other senior professionals within the manufacturing facility. The profile of the respondents is shown in Table 2. Manufacturing enterprises provided the most direct responses to the questions based on their own maintenance strategy, operations, and practices in PHM development and implementation, while technology/consulting companies provided more comprehensive information such as common PHM solutions to various types of industrial sectors.

In particular, the manufacturing enterprises were classified into two general groups based on their sizes: (1) large-sized enterprises, and (2) small and medium-sized enterprises (SMEs). Although there is no universally accepted definition of SME, this study uses the thresholds established by the US Department of Commerce as guidelines, i.e., SMEs are manufacturing enterprises with less than 500 employees and large-sized enterprises with over 500 employees. Moreover, the enterprises represent various sectors within manufacturing, including: automotive, aerospace, transportation, machinery and equipment, consumer products, and electronics.

## 3. Results analysis and discussion

### 3.1. Observations and themes from questionnaire

The questionnaire responses that were collected during the site visits, plant tours, and phone discussions were organized into categorical bins for bar charts. Statistical hypothesis testing was conducted to help summarize the overall sentiment and themes found across the various manufacturing enterprises and technology consulting companies who participated in the study.

According to the survey data, we classify the commonly used measures of maintenance performance into five categories based on their focus. They are:

- measures of equipment performance (e.g., availability, reliability, mean time to failure);
- measures of product quality performance (e.g., defect rate, yield);
- measures of maintenance productivity performance (e.g., manpower utilization, efficiency);
- measures of maintenance cost performance (e.g., maintenance labor and material cost);
- measures of safety and environment (e.g., safety, health and environment incidents).

**Figure 1:**
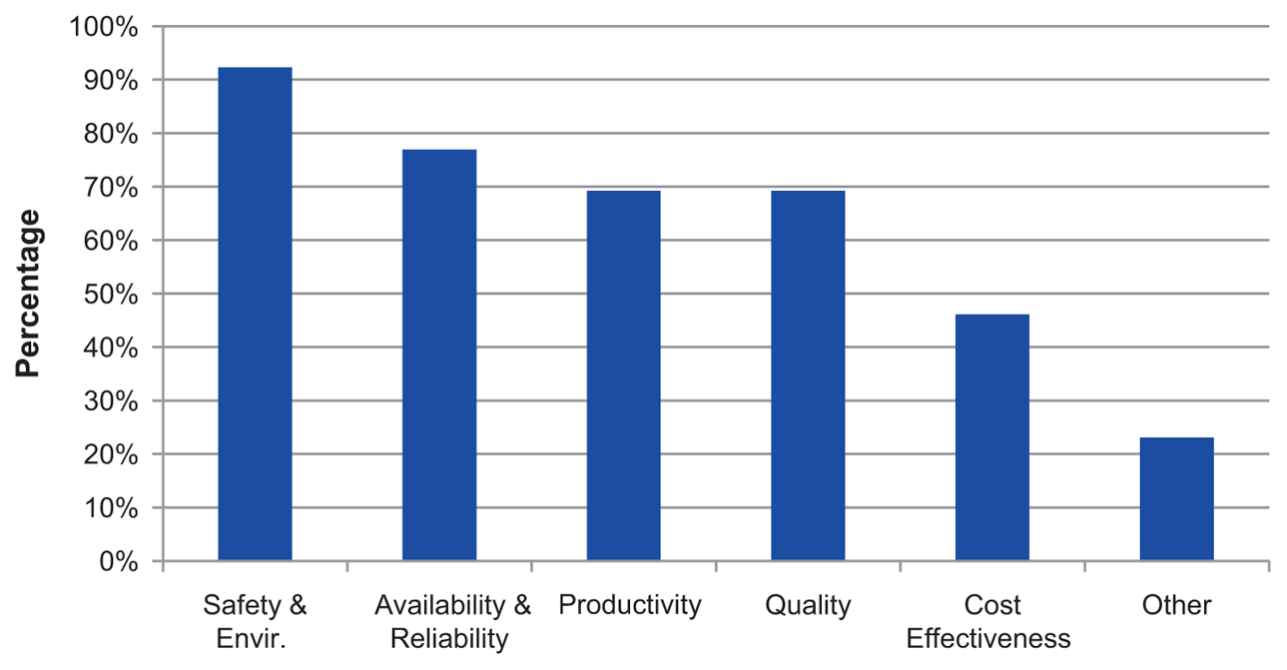
The objective of maintenance pursued by different manufacturing facilities.

Figure 1 shows the percentage of the maintenance objectives pursued by the manufacturing facilities according to the respondents’ selection of important maintenance objectives within their plant.

Several key findings drawn upon this analysis are discussed as follows. First, it was noted that safety, availability, and reliability are the most highly rated maintenance objectives. Productivity and quality are also important maintenance objectives according to 70% of the respondents. It is also noted that with different focuses of manufacturing performance goals, maintenance managers have different objectives of their maintenance function. Second, equipment performance-related indicators are very commonly used. Equipment performance indicators are measured on a shorter time interval (on a daily or weekly basis) than cost performance measures (on a monthly or quarterly basis).

**Figure 2:**
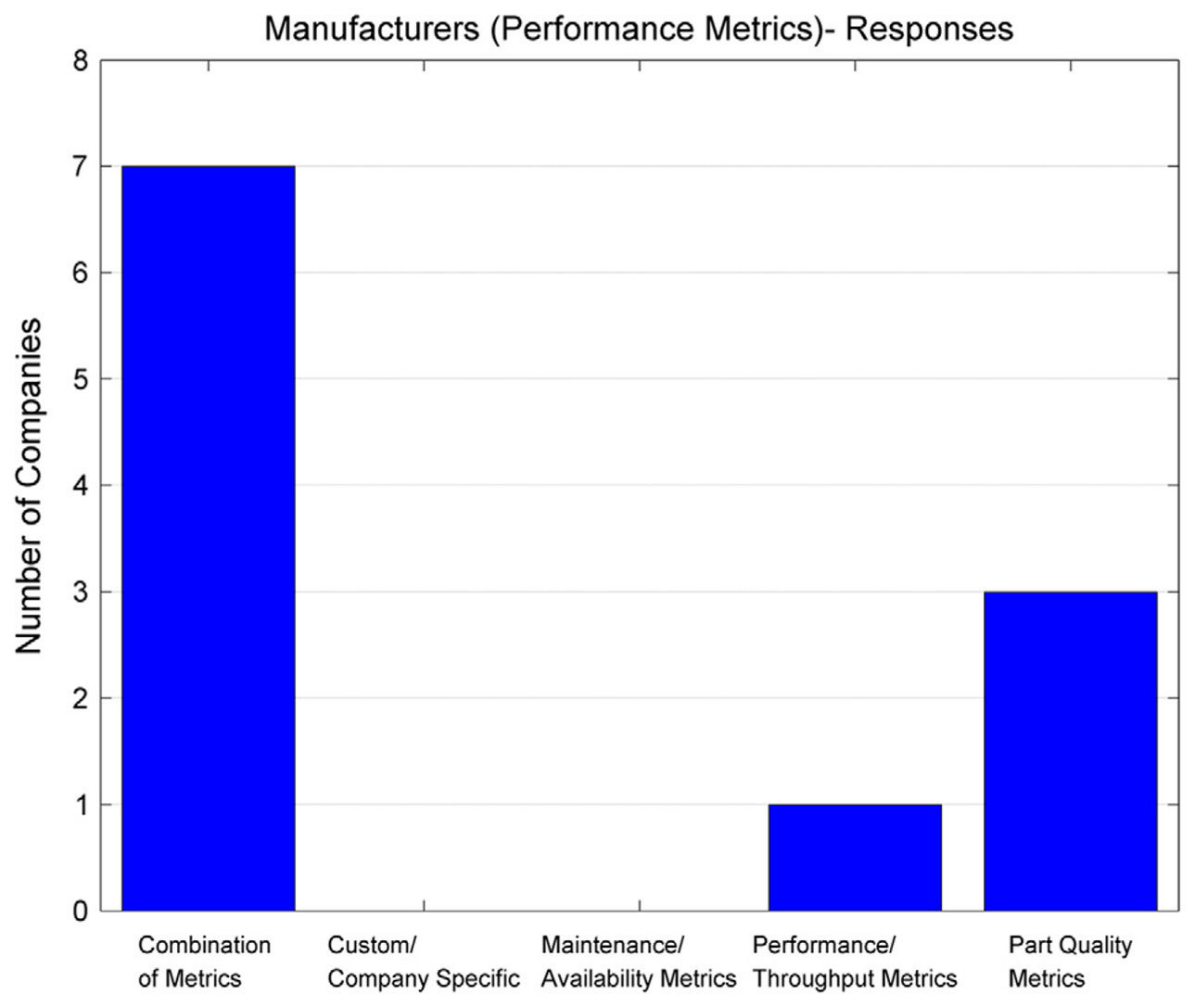
Manufacturers – performance metrics – survey response.

Figure 2 shows the performance metrics used by responding manufacturing organizations, including productivity, maintenance, and product quality. In general, a larger sample size of manufacturing enterprises would be needed to draw more statistically significant conclusions, but it does appear that the majority of the manufacturers surveyed use a combination of metrics (e.g., throughput metrics, part quality metrics, maintenance metrics), shown as the first bar in Figure 2. The idea that a manufacturing enterprise does not rely solely on a single category of metrics (e.g., only part quality or only maintenance) is well aligned with the research about companies adopting the OEE metric to measure their factory performance over time.

**Figure 3:**
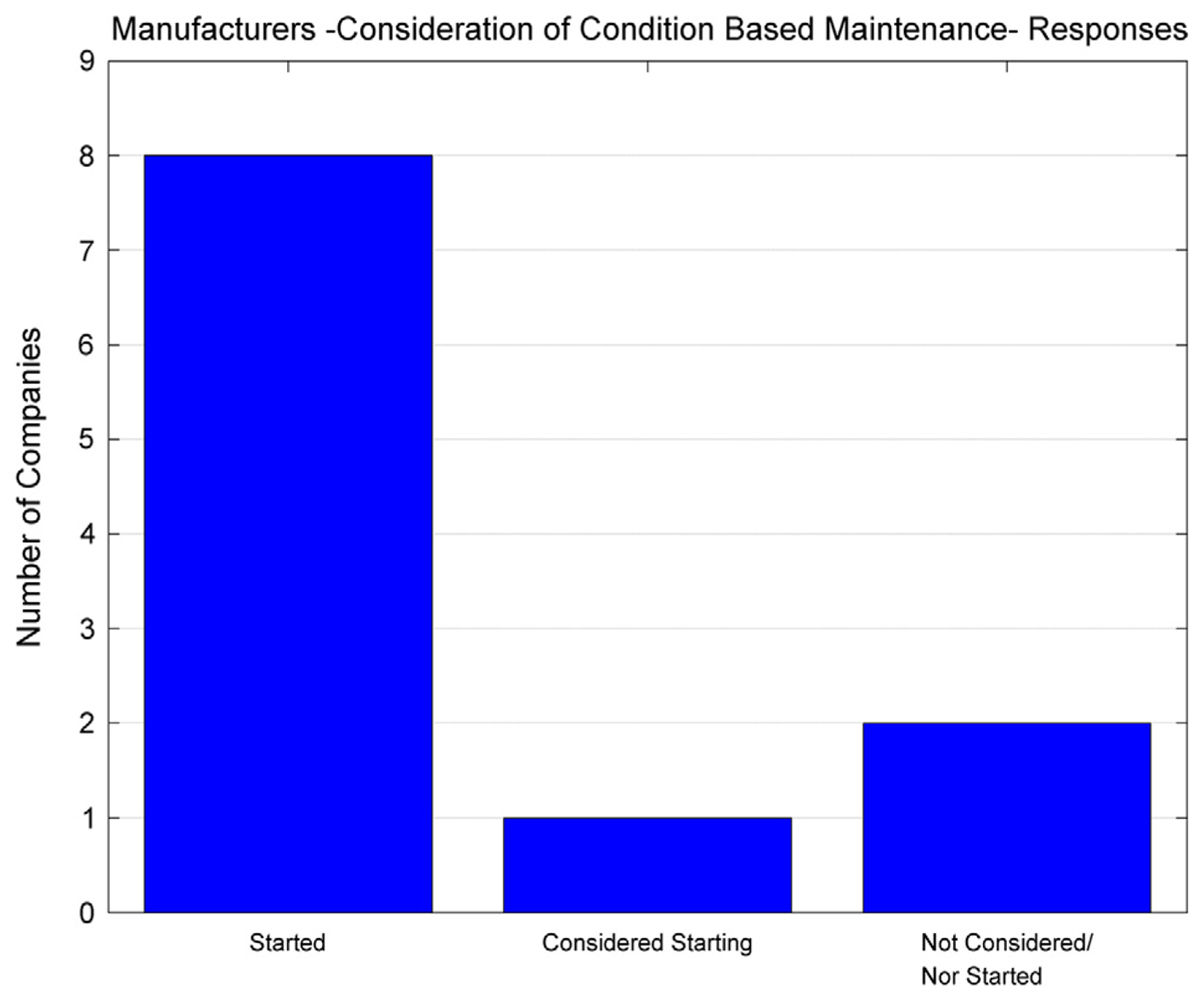
Manufacturers – consideration of condition based maintenance – survey response.

Some important insights were also gained on whether CBM strategies for certain types of machines or processes had been considered by manufacturing organizations surveyed in this study. The responses for this particular question are provided in Figure 3; there appears to be a vast majority of organizations that have started to consider condition-based maintenance approaches.

A Chi-square statistical hypothesis test is performed to see if there is any statistical evidence that the responses to this question (consideration of CBM) are random (null hypothesis) or if there is evidence against that hypothesis. The Chi-square statistical test procedure for categorical variables consists of comparing the expected bin frequencies to the observed bin frequencies. Based on the hypothesis that the responses are random, one would assume an expected frequency count that was even for each bin group (e.g., if there are 20 samples and four bins, one would expect five counts in each bin). The Chi-square test statistic (χ^2^) is given by the expression provided in equation (1) where *n* is the number of bin groups. The degree of freedom for this statistical test is based on the number of bin categories as indicated in equation (2). The results from this statistical hypothesis test are shown in Table 3 in which the test-statistic and *p*-value indicate that there is evidence that the responses are not random. Although the sample size is relatively small, it does provide some general observations that manufacturing organizations are starting to move towards condition-based maintenance strategies.

(1) $$\chi^{2}=\sum_{i=1}^{n}\left(\frac{{\left({\text{Observed}}_{i}-{\text{Expected}}_{i}\right)}^{2}}{{\text{Expected}}_{i}}\right)$$

(2) df=n-1

**Figure 4:**
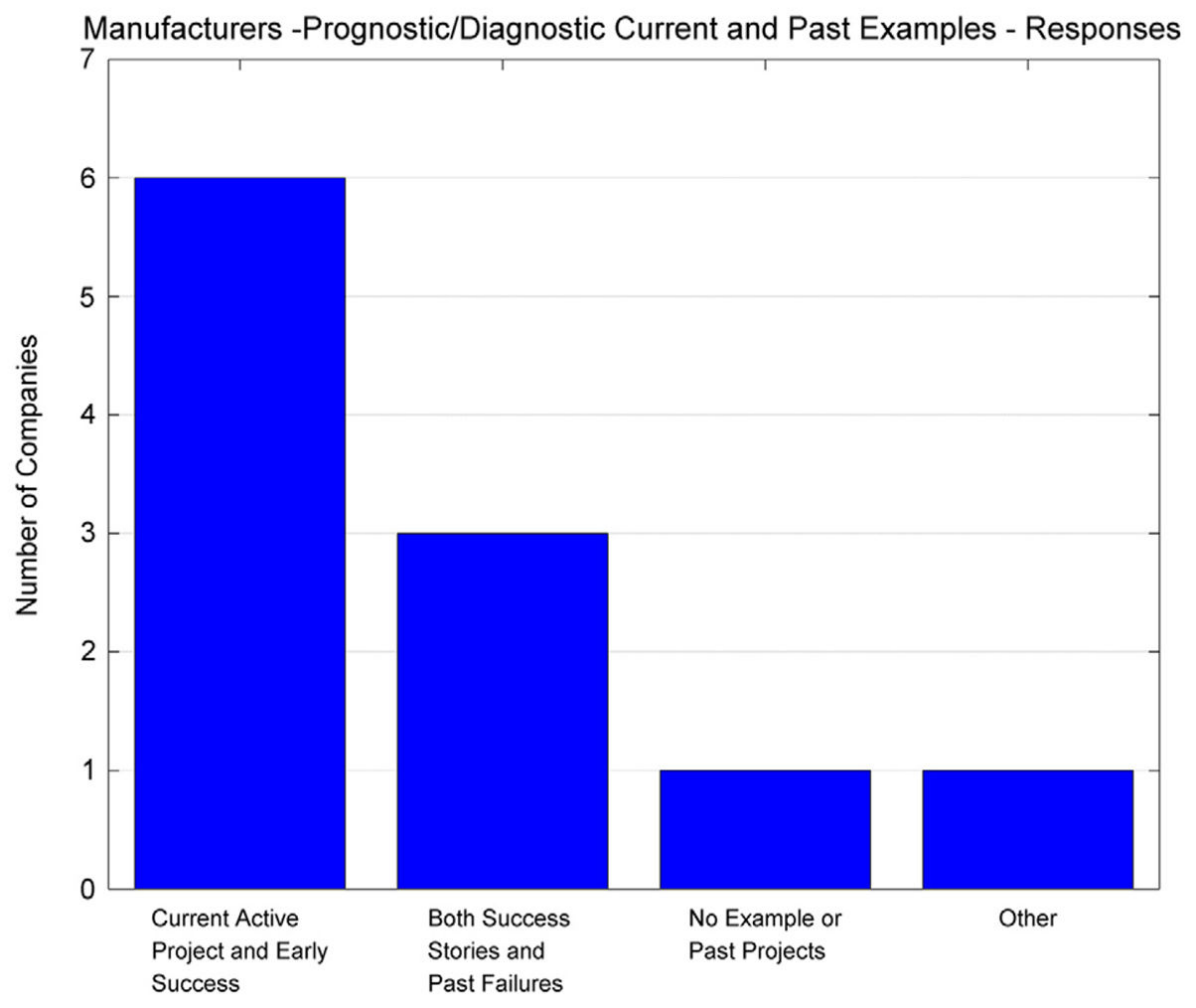
Manufacturers – prognostic/diagnostic current and past examples – survey response.

Although it was observed that companies are starting to consider condition-based maintenance, it was interesting to see if they had current or past diagnostic or prognostic projects. The responses in Figure 4 indicate that a majority of the manufacturing companies had active projects in manufacturing diagnostics and prognostics, while a few had no current projects but had some past projects. It was noted during a conversation with one of the maintenance managers for a construction machine manufacturer that not all of their past diagnostic projects were successful and some project results were disappointing in being overpromised by the diagnostic and prognostic technology.

A Chi-square test was also performed on the bar chart results in Figure 4 to see if there was any evidence that the responses were not random and that the manufacturers had a significant sentiment to a particular response bin. For this question, the Chi-square test statistic was at 6.09 and the *p*-value was also above the alpha level, indicating that there was not enough evidence to reject the null hypothesis that the distribution of the responses was uniform. Perhaps additional manufacturer companies would need to be included in this study to determine if there was any underlying pattern or trend with respect to current or past diagnostic and prognostic related projects (Table 4).

#### Key findings

Most manufacturers are using a combination of metrics that consider part quality, throughput, and maintenance effectiveness. In addition, it was found that the effectiveness of the preventative maintenance programs varied across the manufacturing organizations surveyed. It is postulated that the manufacturing facility size and diversity and age of their assets contributed to the variance in these responses. Also, the vast majority of manufacturing organizations surveyed in this study are starting to consider condition-based maintenance approaches and technology. Many of the manufacturers surveyed had active diagnostic and prognostic projects, although a few mentioned past projects that achieved varying degrees of success. Technology providers and manufacturers had a positive and optimistic viewpoint when considering the future outlook for manufacturing PHM.

### 3.2. Maintenance factors analysis

Building on the questionnaire results presented in Section 3.1, the responses from the manufacturers are further quantified to identify differences between different types of manufacturing organizations. Each factor is divided into three levels, which are advanced, intermediate, and reactive. The eight key factors and the descriptions of three levels of each factor are detailed in Table 5. The three levels presented in Table 5 are explicitly defined for this study to increase the clarity and structure of the responses for the participants.

**Figure 5:**
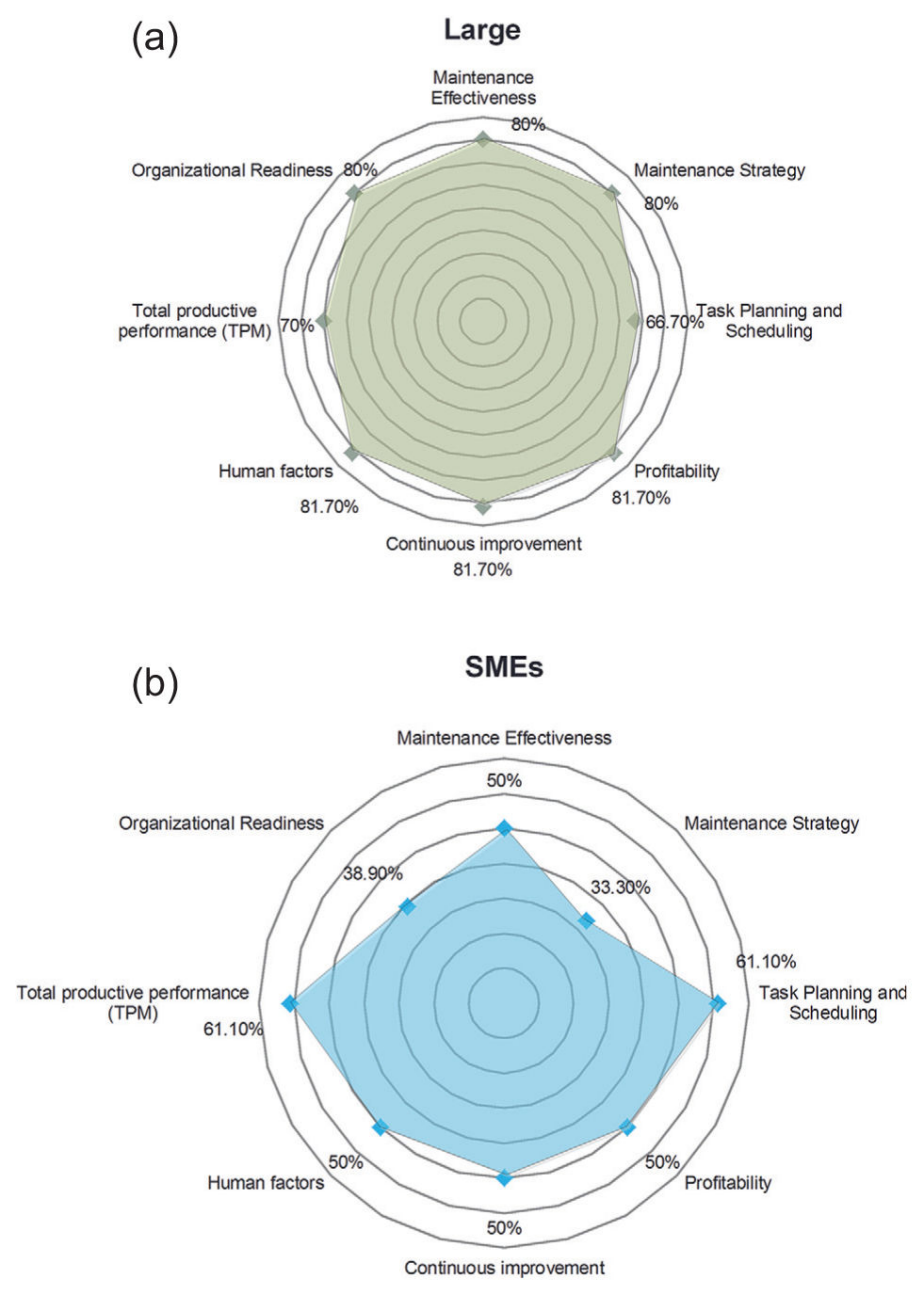
Radar charts for manufacturing enterprises with different sizes: (a) large-sized manufacturing enterprises, (b) SMEs.

In order to study how enterprise size may influence these key factors of maintenance, the responses to the interval questions (based on Table 5) are averaged and plotted in radar charts for large-sized enterprises and SMEs, respectively. Figure 5 presents the average levels of eight key factors for large firms and SMEs. The radar charts display the eight key factors with averaged responses, where the further the data points are from the center, the better performance the maintenance is.

#### Key findings

Some differences can be observed clearly between the two radar charts in Figure 5: company size influences the key factors and the levels of large firms are generally more advanced than SMEs, particularly with respect to the maintenance effectiveness level, maintenance strategy level, profit-ability level, continuous improvement level, human factor level, and organizational readiness level. Meanwhile, the average levels of some other key factors, such as task planning and scheduling level and TPM level, are similar between large firms and SMEs. The radar chart can only present a general comparison. Whether the difference between large firms and SMEs is statistically significant should be further determined by using correlation analysis and hypothesis testing, as seen in the following sections.

### 3.3. Contrasting SMEs and large-sized manufacturers

The correlation analysis indicates that a relationship exists between the size of manufacturing enterprise and the eight key factors; the Student’s *t*-test is adopted to do the hypothesis testing to see whether the differences between SMEs and large-sized manufacturers are statistically significant.

#### Hypothesis testing using Student’s t-test

Due to the small sample number, the Student’s t test is used to check whether there are significant differences in each factor between large manufacturers and SMEs. All eight factors in Table 5 are tested between SMEs and large-sized manufacturers. An example of maintenance strategy level for SMEs versus large firms is presented below to explain how the hypothesis test works.

The null hypothesis on maintenance strategy level *H*_0_ is that the mean maintenance strategy level of SMEs equals the mean maintenance strategy level of large-sized manufacturers. The results of a student’s *t*-test are shown below.

First, Levene’s test is used to check whether the variances of two groups are equal because Levene’s test is an inferential statistic used to assess the equality of variances for a variable calculated for two or more groups. The significance of *F*-value is 0.023, which is less than 0.05, meaning that the variances in the two groups are not equal, i.e., equal variance is not assumed. According to Table 6, two-tailed *t*(0.05, 9) is less than the absolute *t*-value, i.e., |*t*| > *t*(0.05, 9). Therefore, *H*_0_ is rejected, indicating that the mean maintenance strategy level of large-sized manufacturers is significantly larger than the mean maintenance strategy level of SMEs.

Two key findings from the statistical hypothesis tests are: (1) the mean levels of maintenance effectiveness, maintenance strategy, profitability, continuous improvement, HR, and organizational readiness of large-sized manufacturers are significantly larger than those of SMEs; and (2) there is no significant difference between the mean levels of scheduling and TPM of large manufacturers and SMEs.

Overall, the results suggest that large manufacturers, in contrast to SMEs, have the ability to focus on two distinct strategies: (1) continuous improvement on intelligent maintenance technology and quality on one hand, and (2) a combination of low-cost maintenance and innovation on the other hand. This latter strategy is particularly interesting since it denotes simultaneous emphasis on both cost-effective PHM technology innovation and strategy innovation.

### 3.4. Change efforts and barriers

The results also reveal significant differences between SMEs and large companies in their change efforts in improving maintenance practice and PHM technology. Whereas both SMEs and large enterprises are affected by hyper-competition and accelerated pace of change, SMEs appear less able and/or less willing to initiate change in their maintenance functions, mainly because of size-related disadvantages. They are faced with more barriers for change in terms of organizational structure and readiness for innovation and the associated limited finance and human resources.

**Figure 6:**
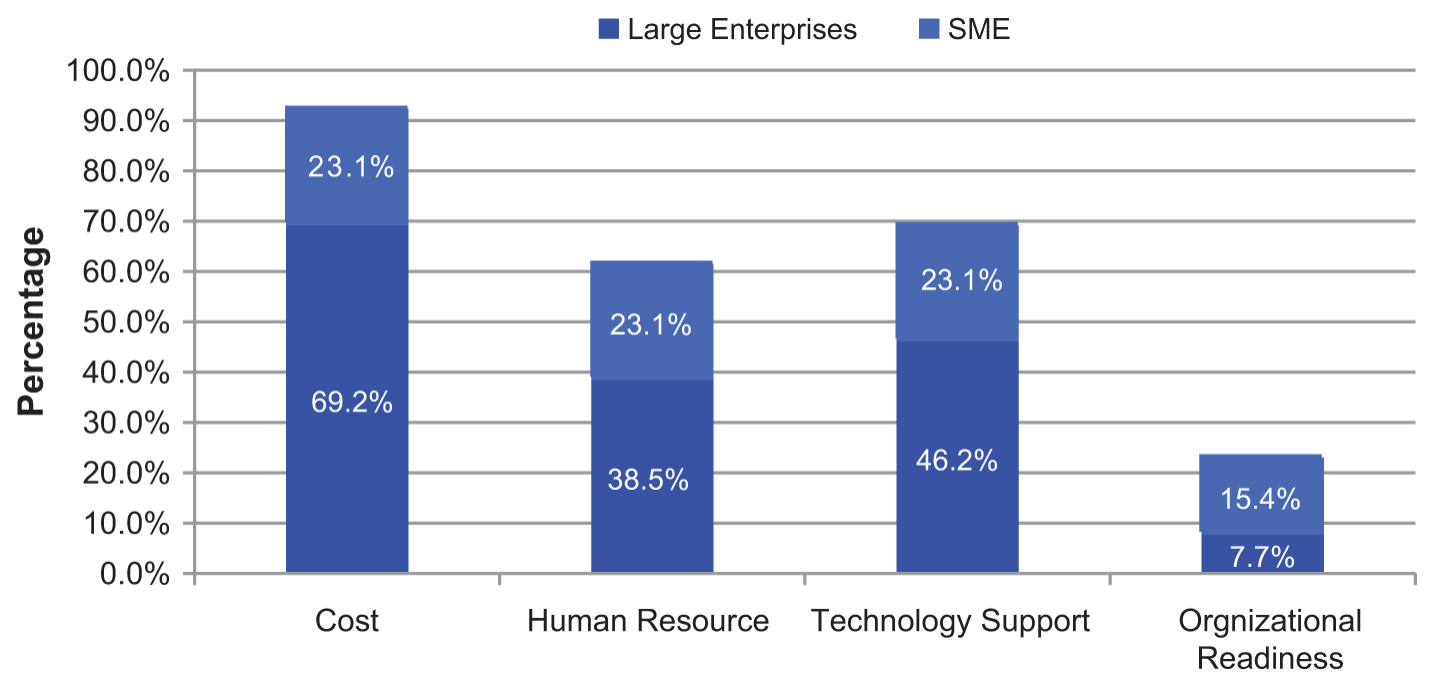
Factors that might be the barriers for companies considering more advanced CBM/PHM technology.

To illustrate the barriers clearly, the respondents’ concerns about future efforts on PHM technologies are presented in Figure 6. Figure 6 shows the factors that might be the barriers for companies considering to change their current maintenance practice and/or invest in more advanced CBM/PHM technologies. Cost is the major concern for both large and small companies. Technical support from a R&D team for CBM and PHM technology implementation is also an important barrier for both but more critical for SMEs.

## 4. Gaps, future trends, and research directions

Some underlying gaps and trends were observed in this study, and these observations will provide some clarity with respect to the research directions for manufacturing PHM. It should be noted that the sample size in this study was small and a larger pool of manufacturers should be considered as an extension to this study to better validate these initial observations and trends that were observed. One of the general observations from this study was that the maintenance strategy level is relatively low, and most manufacturing enterprises willing to improve their maintenance strategies are facing some barriers, such as cost, workforce, technology readiness, system design changes, etc. In addition, large enterprises are making more effort to improve their maintenance strategy because of their size-related advantages such as R&D support and leadership involvement, skilled workforce, and other resources. Besides these more economical barriers for adopting a more condition-based maintenance strategy, the overall state of the art for manufacturing PHM has many current gaps. In particular, the literature survey highlighted that while there is substantial work on component and machine level prognostics and diagnostic research, there is very little prognostics or diagnostic research work that considers multiple machines or a production system. Although it was noted by some technology providers that a system level health monitoring system would be more difficult to achieve, it appears that at least some fundamental research work should be started for the system level diagnostics and prognostics.

In addition, even current machine-level prognostic and diagnostic implementations have current gaps which are limiting its success when implemented by manufacturers. In particular, it seems that more reliable threshold methods and more adaptive machine-level health monitoring models are needed. These would help address some of the manufacturers’ comments on current implementations, which had an unsatisfactory number of false alarms and also had difficulty in collecting baseline data that contains all the possible machine cutting settings and operating modes. It was also noted by the technology providers that the lack of failure data makes it more challenging to develop robust prognostic and diagnostic methods for a variety of reasons. Without reference data sets that include failure data, validation becomes very difficult.

Beyond the technology gaps, there is a lack of buy-in accompanied by low industry-PHM awareness, experience, and training needed to apply principles and tools and what type of value it can bring. To inspire more enterprises, particularly SMEs, more manufacturing PHM case studies should also be presented, whether they document successful or failed PHM attempts. From successful case studies, the enterprises can know how to increase the return on investment using CBM and PHM because the cost is the most significant barrier.

From the failed case studies, the root-cause can be explored. More case studies can help enterprises know exactly how CBM and PHM work, and then, inspire more enterprises to consider CBM and PHM. The case studies should not only focus on the research, but also focus on the cost, i.e., case studies should show customers how CBM and PHM decrease the cost and increase the return on investment. The gaps and barriers for implementing advanced PHM technologies and maintenance strategies in this study agree with the gaps identified in the NIST PHM workshop report [19].

This survey-based pilot study will be used by the stakeholder community to guide future directions for the development of new technologies and infrastructure to support PHM system implementation in smart manufacturing environments. Table 7 shows the research efforts that are expected to address the key elements of future PHM roadmaps at multiple levels.

## 5. Conclusions and future work

This study was aimed at investigating manufacturing industry best practices that the manufacturing industry is currently using to achieve their performance goals by incorporating intelligent maintenance technology and strategies. With that notion in mind, data was collected by phone interviews and on-site facility visits from various manufacturing enterprises, including a total of 15 manufacturing enterprises and eight technology/consulting companies. One of the interesting findings during this study was that the maintenance effectiveness, maintenance strategy, and human resources allocation for maintenance were significantly correlated with the size of the manufacturing enterprise. There was a clear difference in maintenance technology and strategy when comparing large and SME manufacturing enterprises. Even for the larger manufacturing enterprises, it was noted that the effectiveness of their preventative maintenance programs varied between the different organizations and many organizations had mixed success with respect to their past diagnostic and prognostic projects. Despite this mixed level of success, many of the manufacturing organizations surveyed had active diagnostic and prognostic projects and had an overwhelming positive and optimistic viewpoint when considering the future outlook for manufacturing PHM.

The results from this study illustrate many future research directions to address the gaps identified in this study. The literature review highlighted a sparse set of technical work on system-level PHM for factory applications, in comparison to the machine-level and component-level PHM work for robotics, machine tools, and other manufacturing equipment; thus the need to develop technical approaches for system-level PHM for factory applications is one potential future research direction. In addition, some manufacturers were disappointed in the threshold setting and overall robustness in the PHM machine-level models; this reiterates that there is a still a need to improve the current state of the art with respect to PHM for manufacturing components and machines. Lastly, there is a significant gap between SME and large manufacturing organizations, in which the SME would benefit from at least learning from the large manufacturers and their early trials and success with PHM and maintenance technology. With this notion, it would be beneficial to make a concerted effort to disseminate the PHM manufacturing case studies, with the aim that SME’s would eventually consider adopting these maintenance technologies as they see fit.

Besides the gaps and issues being highlighted in this paper, many other issues and challenges that prevent manufacturers from adopting advanced PHM technologies will be further explored and discussed in the future work, such as the need for using digital technologies for data collection and handling and interpreting “industrial big data”, the need to develop protocols and tools to communicate data, information, and metrics across the component, machine and system levels for diagnostics and prognostics in manufacturing, and the need to enhance operations and maintenance intelligence by predictive and preventive control and management.

This survey focused on US-based manufacturing industry. The state of the art and the trend of PHM technologies in the US manufacturing can be related to broader impact on technology development in other countries in Europe and Asia. A large proportion of US manufacturers’ R&D takes place in high technology sectors such as electronics and aircraft manufacturing, whereas in most other counties a far greater portion of manufactures’ R&D outlays occur in medium-technology sections such as motor vehicle and machinery manufacturing. Therefore, R&D spending in PHM technology in the US manufacturing appears to be more important and value added.

Being one of the leading countries in manufacturing, the US will be taking a leading role in the future PHM technology and research development and making broader global impact. Likewise, several of the US manufacturers surveyed have a presence in other countries across the globe. Best practices and lessons learned within a facility inside the US are likely to be disseminated to their foreign counterparts to improve overall productivity and efficiency. In addition to individual companies having footprints across multiple countries, many supply chains span the globe. That is to say a US company that resides within the same supply chain (whether it be upstream or downstream) as a foreign entity is more likely to share their own CBM and PHM best practices and lessons learned if it’s mutually-beneficial. Some of the research needs and directions mentioned above (e.g., smart sensors, data collection and communication; performance metrics, and assessment; and work-force skill and training) would also bring tremendous value to the international community with their input and involvement. Supply chains are so interconnected on a global scale that a fault or failure at a specific node in the supply chain can cascade beyond the home country of the fault. Given this phenomena, one can theorize that enhancing one’s CBM and PHM practices and disseminating these findings to its collaborators can have a positive cascading effect.

## Figures and Tables

**Table 1:** Maintenance strategy evolution.

Maintenance strategy	Reactive maintenance (RM)	Preventive maintenance (PM)	Predictive maintenance (PdM)	Prognostics & health management (PHM)
Maintenance interval	Fail and fix	Time based; usage based	Reliability based; condition based	Improve & sustain
Object	Component	Component; function;	Component; function; system	Component; function; system
Planning & Scheduling	Planning on the fly	Planning & scheduling based on optimal PM interval	Predictive planning & scheduling	Proactive planning & scheduling
Failure severity and frequency	Low severity, low frequency	Low to medium severity, high frequency	Medium to high severity, low frequency	High impact, high frequency
Human factor (inspection & decision-making)	High	Intermediate	Low	Low (false alarm)
Cost effectiveness	Labor intensive; Labor and material	Costly due to over maintenance or ineffective & inefficient PM	Cost-effective; extended life & less failure-induced costs	Cost-effective: Substantially save failures & extend the life of equipment
Requirement for technology readiness	Low	Low	High	High

**Table 2:** Participating enterprises segmented by size and type.

	SME	Large	Total	Percentage
Manufacturing	3	12	15	65.2
Technology/consulting	5	3	8	34.8
Total	8	15	23	100

**Table 3:** Chi-of condition based maintenance.

χ^2^	7.8182
α	0.05
*df*	2
*p*-value	0.0201
Hypothesis	*H*_a_

**Table 4:** Chi-square test results – consideration of condition based maintenance response.

χ^2^	6.0909
α	0.05
*df*	3
*p*-value	0.1073
Hypothesis	*H*_0_

**Table 5:** Key factors versus maintenance performance at various levels.

Key factors	Level 3 (100%) advanced	Level 2 (66.7%) intermediate	Level 1 (33.3%) reactive
Maintenance effectiveness	Maintenance performance is very satisfactory.	Maintenance program is effective but could still be improved.	Maintenance has significant room for improvement, or preventive maintenance program is lacking/reactive maintenance.
Maintenance strategy	Employ predictive maintenance (PdM) strategy for sustainable improvement. All problems are analyzed and permanently solved.	Use preventive maintenance (PM) as a main approach, usually age-based or cycle-based.	Rely heavily on reactive maintenance (RM), no equipment health information involved.
Task planning and scheduling	More than 90% of work that is planned is accomplished. Low overtime for maintenance activities (<15%).	More than 50% work planned accomplished. Relatively high overtime (>15%).	Less than 50% work planned accomplished. High overtime (>30%).
Profitability	Significant cost saving due to failure reduction and life extension.	Cost-effectiveness is satisfactory.	Not cost-effective.
Continuous improvement	Proactive maintenance. CBM or PHM applied, performance measurements are in place and effectively used.	Have PM in place with management involved in policy settings and reviews.	Have no CBM or PHM. Low involvement of management. Reactive maintenance is very common.
Maintenance training	Educational plans for each maintenance worker. A global R&D team that is responsible for developing and implementing prognostic and diagnostic techniques.	Skilled staff normally qualified on a few machines. Small team that is responsible for developing and implementing prognostic and diagnostic techniques.	No training on how to use PM or other maintenance strategies. Lack of system to collect maintenance knowledge. No team that is responsible for developing and implementing prognostic and diagnostic techniques.
Total productive performance (TPM)	Overall Equipment Effectiveness (OEE) is greater than 80%.	Overall Equipment Effectiveness (OEE) is between 50% and 80%.	Overall Equipment Effectiveness (OEE) is less than 50%.
Organizational readiness	Leadership involvement R&D support.	Lack of sufficient R&D support & leadership involvement.	“Fire Fighting” approach.

**Table 6:** Student’s *t*-test for maintenance strategy comparison between large enterprises and SMEs.

	Levene’s test for equality of variances		*t*-test for equality of means		
	*F*	Significance	*t*	*df*	Significance (2-tailed)
Equal variance assumed	6.913	0.023	−5.961	11	0.000094
Equal variance not assumed			−11.225	9	0.000001

**Table 7:** Priority roadmaps of research effort.

Categories of PHM roadmaps	Priority of research effort	
	Component/machine level	System level
Diagnostics & PHM technology and metrics		
Smart sensors, data collection & communication	High	Medium
PHM algorithm design and analysis	High	Medium
Software, hardware and integration for PHM	Medium	High
Maintenance strategy		
Performance metrics and assessment	Medium	High
CBM and PHM based maintenance scheduling	Medium	High
Organizational readiness		
Workforce skill & training	High	Medium
R&D support & leadership	Medium	High
Analysis of cost, complexity & risk of adopting new PHM	Medium	High
